# Combined ultrasonography and CT for prognosis and predicting clinical outcomes of patients with pseudomyxoma peritonei

**DOI:** 10.1007/s00330-022-09242-z

**Published:** 2022-11-23

**Authors:** Xuedi Han, Qian Zhang, Nan Zhou, Ruiqing Ma, Jiajun Wang, Xichao Zhai, Bin Cui, Yiyan Lu, Lei Liang

**Affiliations:** 1 Department of Ultrasound, Aerospace Center Hospital, No. 15 Yuquan Road, Haidian District, Beijing, 100049 China; 2 Department of Gastroenterology, Beijing Friendship Hospital, Capital Medical University, No. 95 Yong’an street, Xuanwu District, Beijing, 100050 China; 3 Department of Myxoma, Aerospace Center Hospital, No. 15 Yuquan Road, Haidian District, Beijing, 100049 China; 4 Department of Radiology, Aerospace Center Hospital, No. 15 Yuquan Road, Haidian District, Beijing, 100049 China; 5 Department of Pathology, Aerospace Center Hospital, No. 15 Yuquan Road, Haidian District, Beijing, 100049 China

**Keywords:** Pseudomyxoma peritonei, Peritoneal cancer index, Prognosis, Ultrasonography, Tomography, x-ray computed

## Abstract

**Objectives:**

This study aimed to identify the diagnostic accuracy of combined ultrasonography (US) and computed tomography (CT) in evaluating the tumor burden of pseudomyxoma peritonei (PMP). Besides, we assessed the ability of this combination to predict the likelihood of complete resection.

**Methods:**

This retrospective study involved 504 patients diagnosed with PMP and scheduled for cytoreduction surgery. We compared tumor burden—quantified as peritoneal cancer index (PCI) by preoperative US and CT (US-CT-PCI)—with surgical findings. Next, we assessed the prognostic value of US-CT PCI and imaging features in determining the completeness of cytoreduction (CCR) score using multivariate analysis.

**Results:**

US-CT PCI demonstrated a high PCI evaluation accuracy under moderate tumor burden. Higher US-CT PCI could predict incomplete resection. In addition, we identified imaging features such as mesenteric involvement as an independent predictor of incomplete resection (hazard ratio (HR) = 2.006; *p* = 0.007).

**Conclusions:**

US-CT PCI allowed us to predict the completeness of cytoreductive surgery in patients with PMP. Moreover, the combined US and CT imaging detected several features indicating incomplete cytoreduction.

**Key Points:**

*• Ultrasonography (US) can act as a complementary diagnostic modality in peritoneal cancer index (PCI) evaluation by combining CT in the small bowel area and US in the abdominal area.*

*• A modified peritoneal cancer index (US-CT PCI) helps preoperatively evaluate tumor burden with high accuracy and allows to predict incomplete resection.*

*• US-CT PCI of 20 or above and the involvement of particular structures such as mesentery, independently indicate incomplete resection.*

**Supplementary Information:**

The online version contains supplementary material available at 10.1007/s00330-022-09242-z.

## Introduction

Pseudomyxoma peritonei (PMP) is a rare clinical condition most commonly originating from the perforated epithelial neoplasm of the appendix. Cytoreductive surgery (CRS) combined with hyperthermic intraperitoneal chemotherapy (HIPEC) has been recently advocated as the mainstay treatment for PMP [1]. It provides optimal outcomes in patients with PMP [2, 3]. When complete CRS is not feasible, debulking surgery can also improve PMP symptoms [4]. The surgical peritoneal cancer index (S-PCI), obtained by surgical exploration, helps predict the outcomes and expect benefits [5]. However, major adhesions from previous surgery often hinder thorough exploration [6]. Besides, considering the possibility of surgical morbidity, CRS candidates should be carefully selected [5, 7]. Thus, preoperative imaging would improve preoperative planning and prevent unnecessary surgeries.

MRI is increasingly recognized as an alternative imaging modality [8, 9]. It allows to effectively assess subtle small bowel involvement and hepatic hilum invasion, which are two important resectability risk factors [10, 11]. Computed tomography (CT) remains the optimal mode of routine preoperative imaging, it allows the non-invasive evaluation of PCI [12]. However, CT may underestimate the true PCI due to insufficient soft tissue contrast [13]. CT enteroclysis surpasses conventional CT in detecting mucosal and wall abnormalities in the small bowel. Meanwhile, the predictive power of ultrasonography (US) remains under-investigated. As the first modality for investigating unclear abdominal problems, it clearly reveals the most common PMP features, such as ascites and omental cake [14].

We compared the incomplete cytoreduction outcomes’ prediction accuracy of CT alone with combined US and CT. Besides, we evaluated the predictive value of CT and US features for incomplete cytoreduction.

## Materials and methods

### Study population and follow-up

The Aerospace Center Hospital Ethics Committee approved the retrospective study. We performed it in accordance with the Helsinki declaration and its later amendments or comparable ethical standards.

We collected data from all the patients scheduled for CRS treatment in our hospital between April 2008 and Dec 2018. The inclusion criteria were as follows: (1) all patients with a confirmed PMP diagnosis; (2) patients who underwent both US and CT examination 1–2 weeks before surgery; (3) patients with available S-PCI and pathological results. The exclusion criteria were patients with exploratory laparotomy only. We also collected age, gender, and primary tumor site data.

The current PMP classification consensus divides patients into three categories: “low-grade,” “high-grade,” and “with signet ring cells” [15]. Here, we used two categories, low-grade and high-grade, and included patients “with signet ring cells” in the latter. Follow-up abdomen CT scans every 6 months for the first 5 years, then once a year in the following years were advised. All patients provided informed consent.

### Preoperative imaging and US-CT PCI evaluation

We performed preoperative CT scans and US examinations using a Light Speed VCT apparatus and an Aixplorer Ultrasound diagnostic System, respectively. We conducted bowel preparation instead of enteroclysis. Patients had to fast for 8 h before the examination. The patients received 500 mL of 3% meglumine diatrizoate orally at 9 pm on the previous day, 2 h before the exam, and 10 min before the exam. The CT scan was performed from the top of the diaphragm to the plane of the symphysis pubis in the supine position. We first conducted the CT plain film scanning with a tube voltage of 120 kV, tube current of 250 mA, and reconstruction layer thickness and interval of 0.625 mm. Next, we performed enhanced CT scanning after intravenous injection with non-ionic contrast media (iopromide). Two senior specialists independently examined all the US and CT images and reported the classical features of liver scalloping, ascites, abdominal lymph nodes, omental cake, mesenteric involvement, hepatic hilum involvement, small omentum involvement, and abdominal mass.

We assessed the extent of the peritoneal involvement using Sugarbaker’s PCI, which divides the abdomen into nine anatomical areas (Region (R) 0–8) and four small bowel areas (R9–12) [16]. Thus, we divided US and CT readings according to the body surface markers. We obtained the nine abdominal areas by tracing horizontal lines from the lowest rib points and the anterior superior spine on both sides and two extended mid-clavicular vertical lines. The umbilical region, right hypochondrium, epigastric region, left hypochondrium, left lumbar, left iliac region, hypogastrium, right iliac region, and right lumbar correspond to R0–8 in US and CT scans. We located R9–12 in CT by tracking intestine motility. Meanwhile, in US, we located R9–12 by the connected structure of the duodenum and ileocecal region separately and R10–11 by the intestinal distribution.

We gave each abdominal site a score from 0 (no tumor) to 3 (lesion size > 5 cm). We ranked the disease stage as low volume (PCI range 0–9), moderate volume (PCI range 10–19), or high volume (PCI of 20 or above) [17]. We estimated a preoperative US PCI and CT PCI for each patient. PCI evaluation by combining US and CT (US-CT-PCI) combined US PCI in R0-8 and CT PCI in R9-12. Using Spearman correlation, we compared the relationship between US and CT PCI with S-PCI in each region. We assessed the reproducibility of two radiologists in US PCI and CT PCI in each patient using the intra-class correlation coefficient. We only measured the omental cake score for region 0; if we observed signs of visceral scalloping or omental cake, the score was 3 for region 1 or 0, respectively. Otherwise, the lesions—including mesenteric involvement, lesions in lesser omental bursa or porta hepatis, and abdominal mass—were scored in the corresponding regions by size. These examinations were all conducted by one doctor with 10 years of experience in PMP examination. The saved images were reviewed and scored by two radiologists. In cases of disagreement, the final consensus was achieved by discussion. The evaluators did not know the CT PCI results during US PCI scoring, and vice versa.

### Surgery details and S-PCI evaluation

CRS aimed to remove all visible disease by greater omentectomy-splenectomy, left upper quadrant peritonectomy, right upper quadrant peritonectomy, lesser omentectomy-cholecystectomy and pelvic peritonectomy with resection of the sigmoid colon, and antrectomy according to the clinical condition and structures involvement [18]. Segmental obstruction or tumor interaction of the small intestine and tumor nodules larger than 5 cm on the small intestine surface could affect resectability. The S-PCI was scored and calculated during the operation according to Sugarbaker’s published techniques.

### Operative disease clearance evaluation and HIPEC

We assessed the completeness of cytoreduction (CCR) score immediately at the completion of the CRS to evaluate the extent of complete cytoreduction. We considered CCR scores of 0/1 (CCR-0 or CCR-1) (no visible tumor nodules or residual tumor nodules smaller than 2.5mm) as adequate cytoreduction and CCR-2/3 (residual tumor nodules ≥ 2.5 mm) as incomplete cytoreduction [7].

We delivered intra-operative hyperthermic chemotherapy immediately after cytoreduction with a target temperature of 43 °C. We performed HIPEC with Mitomycin C (20 mg/m^2^) or Cisplatin (60 mg/m^2^) delivered during a 90-min thermal cycle. Patients with renal failure, old age, or major co-morbidities received reduced doses. Because of the renal toxicity of both Cisplatin and Mitomycin C, patients with impaired renal function were suggested to be treated at a reduced dose. Cisplatin with a decrease of 50% was applied in patients with creatinine clearance (CrCl) of 30–60 mL/min. Mitomycin C with a decrease of 25% was suggested in patients with CrCl of 10–60 mL/min. HIPEC was recommended in all patients except those with poor healing factors, including high anastomotic tension, intestinal obstruction, and unstable vital signs.

### Statistical analysis

We performed pair-wise comparisons of US-CT PCI and CT PCI in sensitivity and specificity among different imaging features and tumor burdens. We conducted univariate and multivariate analyses to find independent factors of incomplete cytoreduction. All the PMP imaging features were involved. Besides, the univariate analysis of incomplete cytoreduction prediction took into account sex, age, pathology grade, and HIPEC. Factors showing statistical significance in the univariate analyses were evaluated in the multivariate analyses.

We performed multivariate analyses with Cox regression models to identify independent predictors of CCR-2/3. We performed all analyses using SAS and SPSS software. A *p* value < 0.05 was considered statistically significant.

## Results

### Demographic characteristics

**Fig. 1:**
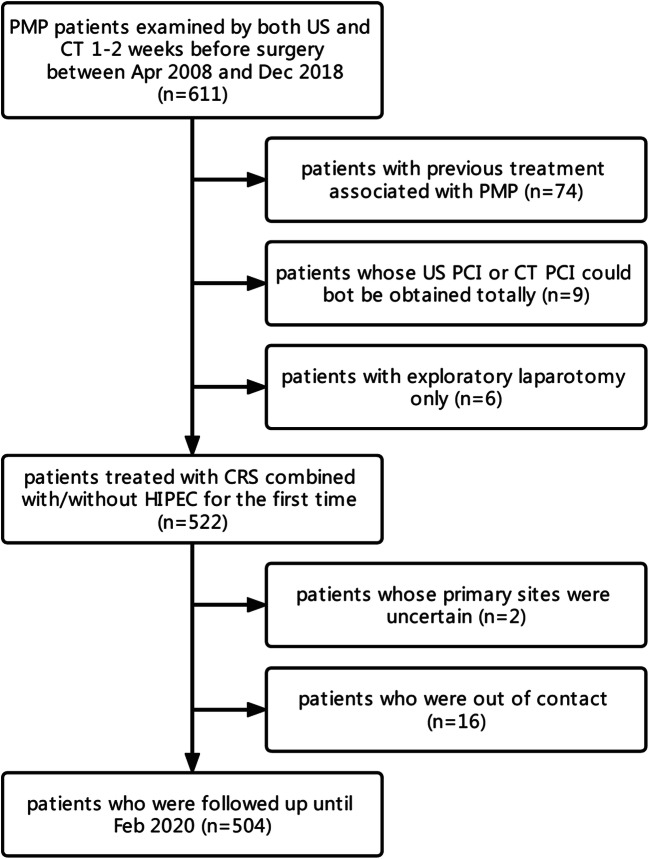
Flow chart of patient selection

In total, we examined 611 PMP patients by both US and CT 1–2 weeks before CRS in the Aerospace Center Hospital. We excluded 74 patients with previous treatment associated with PMP. We also excluded six patients with exploratory laparotomy only and nine with incomplete images for PCI evaluation. Finally, we excluded two patients with uncertain primary sites and 16 who did not attend follow-up sessions. Ultimately, 504 patients were included, with a mean age of 56 years (range 19–85 years) (Fig. 1).

Table 1 shows the patients’ demographics and disease characteristics. There were 186 male and 318 female. There were 103 patients with CCR-0 and 147 with CCR-1. Among the 254 patients with CCR-2/3, 195 did undergo HIPEC. We identified 349 (69.2%) patients as low-grade PMP and 155 (30.8%) as high-grade PMP, including 40 cases as signet ring cells. In 95.63% of these patients, PMP was of appendiceal origin; other origins included colorectum (nine cases), ovary (five cases), digestive tract (three cases), gyneduct (two cases), urachus (two cases), and pancreas (one case). US-CT PCI evaluations revealed that 107 patients had low tumor loads (PCI range 0–9), 69 had moderate tumor loads, and 328 had high tumor loads.

**Table 1:** Demographic and clinical characteristics, and PCI evaluation of patients

Characteristics		N	(%)
Age		56.33 ± 11.68	
Gender	Female	318	63.1
	Male	186	36.9
CCR^a^	0/1	250	49.6
	2/3	254	50.4
HIPEC^b^	Yes	438	86.9
	No	66	13.1
Pathology	Low–grade	349	69.25
	High–grade	155	30.75
S-PCI^c^	0–9	102	20.24
	10–19	60	11.90
	≥ 20	342	67.86
US-CT PCI^d^	0–9	107	21.23
	10–19	69	8.33
	≥ 20	328	70.44
US PCI	0–9	112	22.22
	10–19	119	23.61
	≥ 20	273	54.17
CT PCI	0–9	107	21.23
	10–19	41	8.135
	≥ 20	356	70.635

Note: ^*a*^*CCR*, completeness of cytoreduction; ^*b*^*HIPEC*, hyperthermic intraperitoneal chemotherapy; ^c^*S-PCI*, surgical peritoneal cancer index; ^d^*US-CT PCI*, peritoneal cancer index evaluation by combining ultrasonography and computed tomography

The new classification of surgical complications considers grade III–IV complications as major complications [19]. In our study, 41 (8.13%) patients experienced major complications, and 3 succumbed to perioperative death. Besides, 13 patients experienced infectious complication, including intra-abdominal (*n* = 8) and wound (*n* = 5) infections. Twelve patients experienced intestinal fistula, two had urinary leakage, and five had intra-abdominal hemorrhage.

### Comparing US and CT for PCI evaluation

**Fig. 2:**
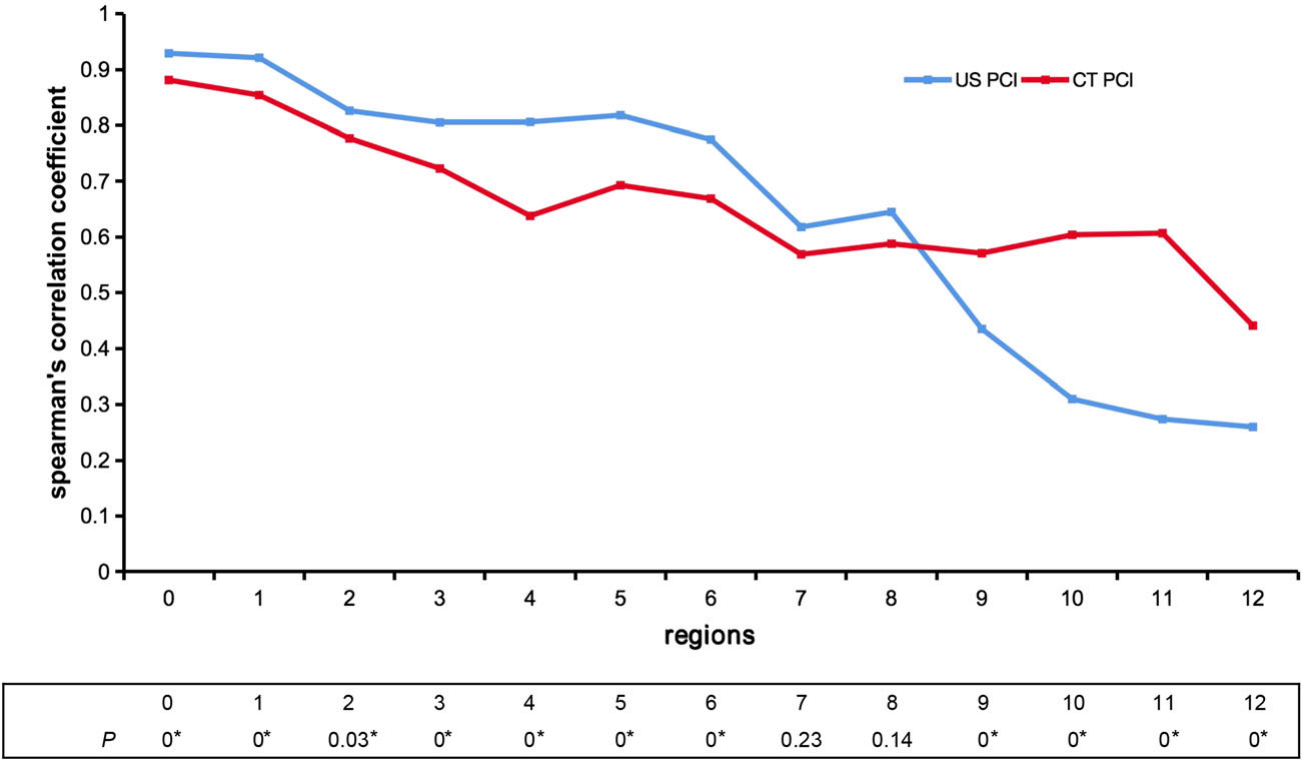
The Spearman correlation analyses between US-CT PCI and S-PCI in 13 abdominopelvic regions. The coefficients of US PCI were high in the nine frontal regions (R0–8) but fell to less than 0.4 (0.25–0.37) in the four other regions (R9–12). The coefficients of CT PCI fluctuated around 0.5 but decreased from the nine frontal regions to the four other regions. The US PCI had higher diagnostic accuracy than CT PCI in seven of the nine abdominal regions (including R0–6) and slightly lower than CT PCI in two of the four small bowel regions. The differences between US PCI and CT PCI were significant in all the regions except R7 and R8

The median US PCI was 17.13 (range 0–31), median CT PCI was 21.42 (range 0–39), and the median S-PCI was 22.45 (range 0–39). The Spearman correlation analyses of US-CT PCI and S-PCI in 13 different abdominopelvic regions (Fig. 2) revealed significant differences between US PCI and CT PCI in all the regions except R7 and R8. The US PCI coefficients were higher than those of CT PCI in the nine frontal regions. Although the CT PCI coefficients decreased from the first to the last region, they were higher than those of US PCI in R9–12.

### Application of combined imaging modalities in disease burden quantification

We evaluated the combined application of US PCI in the nine abdominal regions and CT PCI in the small bowel regions. We defined positive results obtained by US or CT as true positives of US-CT and negative results obtained by both US and CT as true negative results. The US-CT PCI was more consistent with S-PCI than CT PCI (0.796, 0.741). The US-CT results were superior to those of CT alone in patients with US-CT PCI of 10–19 (*p* = 0.021) (Table 2). As shown in Table 3, all the common PMP features in US-CT had a higher area under the curve than in CT alone (*p* < 0.05).

**Table 2:** Comparison of the PCI diagnostic accuracy of US-CT PCI and CT PCI among the three subgroups (PCI 0–9, 10–19, ≥ 20)

	Sensitivity		Specificity		AUC^a^		*p* value for AUC of US-CT^b^ vs CT
	CT	US-CT	CT	US-CT	CT	US-CT	
S-PCI^c^: 0–9	93.14%	95.10%	97.01%	97.51%	0.951	0.963	0.238
S-PCI: 10–19	44.12%	61.76%	96.85%	93.24%	0.709	0.791	0.021
S-PCI: ≥ 20	96.78%	92.11%	84.57%	91.98%	0.907	0.920	0.285

Note: ^*a*^*AUC*, area under the curve; ^b^*US-CT*, combined preoperative ultrasonography and computed tomography; ^c^*S-PCI*, surgical peritoneal cancer index

**Table 3:** Comparison of the detection accuracy on imaging features between combined imaging and CT alone

	Sensitivity		Specificity		AUC^a^		*p* value for AUC of US-CT^b^ vs CT
	CT	US-CT	CT	US-CT	CT	US-CT	
Ascites	91.12%	97.13%	64.52%	64.52%	0.778	0.808	< 0.001
Abdominal lymph nodes	100.00%	100.00%	80.76%	78.99%	0.904	0.895	0.014
Liver scalloping	92.53%	98.45%	81.03%	79.31%	0.868	0.889	< 0.001
Omental cake	58.84%	93.54%	84.76%	84.76%	0.718	0.892	0.007
Hepatic hilum involvement	87.74%	100.00%	78.49%	50.62%	0.804	0.753	< 0.001
Mesenteric involvement	68.10%	74.29%	100.00%	84.01%	0.841	0.792	< 0.001
Small omentum involvement	78.99%	100.00%	52.63%	45.49%	0.658	0.727	< 0.001
Abdominal mass	84.21%	91.50%	32.30%	32.30%	0.583	0.619	< 0.001

Note: ^*a*^*AUC*, area under the curve; ^*b*^*US-CT*, combined preoperative ultrasonography and computed tomography

The variability of US image scanning makes repeatability challenging. In our center, radiologists must be aware of the most common PMP findings [20] and common locations. The diagnosis and PCI evaluators were trained on images of 100 patients, and then their diagnostic accuracy was tested on 100 other patients (Supplementary Figure 1). Radiologists who had a diagnostic accuracy higher than 0.8 became investigators. The two radiologists involved in this study had good reproducibility (intra-class correlation coefficient = 0.863).

### Predictive factors of incomplete cytoreduction

**Fig. 3:**
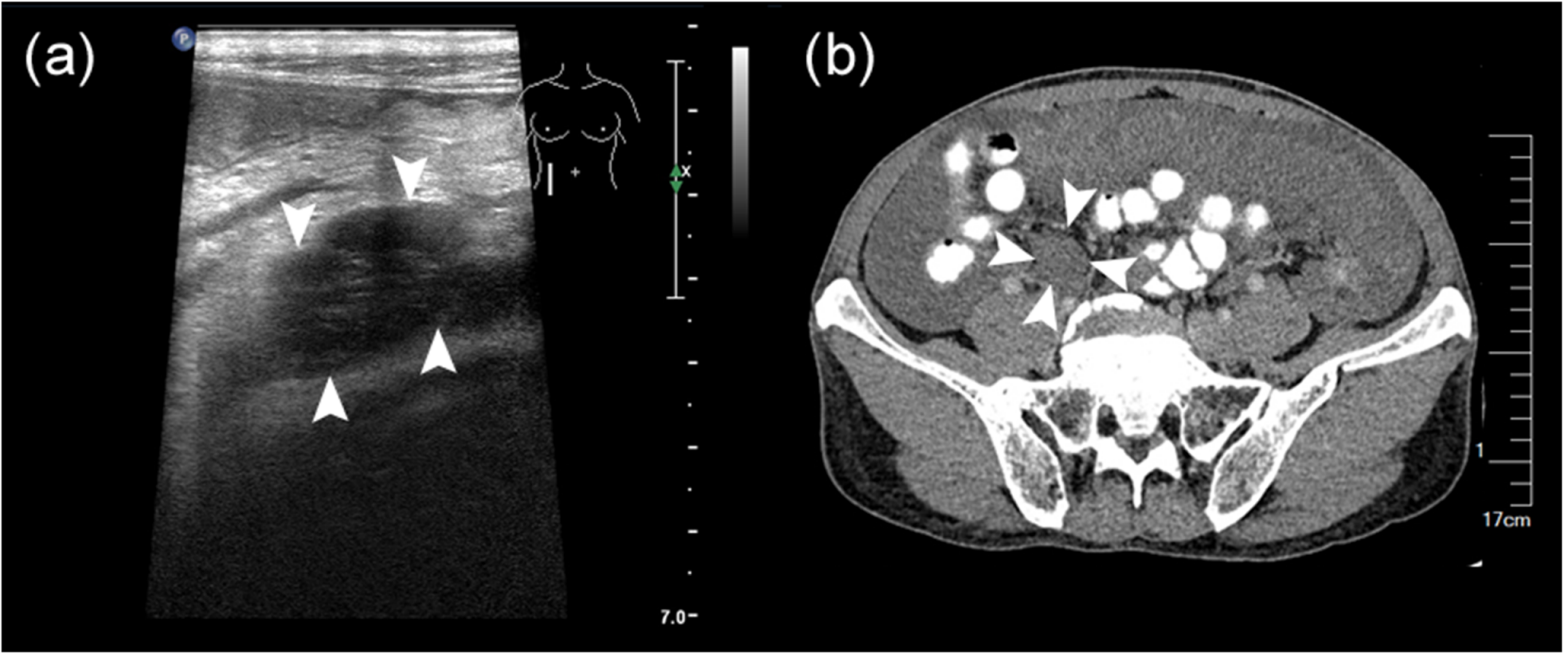
The typical imaging features in US and contrast CT. **a** and **b** showed mesenteric involvement in US and CT

A high US-CT PCI score was a significant predictor of unresectability (PCI ≥ 20; hazard ratio (HR) = 57.281; *p* = 0.002). The analysis demonstrated that mesenteric involvement (Fig. 3) was superior to other imaging features for predicting incomplete cytoreduction. Having mesenteric involvement (HR = 2.006; *p* = 0.007) correlated to the likelihood of achieving inadequate CRS. Gender (HR = 2.271; *p* = 0.002) was also independent predictor of incomplete cytoreduction (Table 4).

**Table 4:** The multivariate logistic regression for incomplete cytoredction by clinical factors and imaging features preoperatively detected by US-CT

		Univariate logistic regression analysis			Multivariate logistic regression analysis		
		Hazard ratio	95% CI^a^	*p* value	Hazard ratio	95% CI	*p* value
Age		1.013	0.997–1.028	0.106			
Gender	Male vs Female	1.576	1.094–2.269	0.015	2.271	1.354–3.811	0.002
US-CT PCI^b^	0–9	Reference			Reference		
	10–19	29.434	3.788–228.694	0.001	9.469	0.789–113.682	0.076
	≥ 20	280.207	38.545–>999.999	< 0.001	57.281	4.611–711.613	0.002
Ascites	Yes vs No	95.831	23.310–393.980	< 0.001	4.275	0.638–28.661	0.135
Abdominal lymph nodes	Yes vs No	5.995	3.998–8.991	< 0.001	1.528	0.911–2.565	0.108
Liver scalloping	Yes vs No	51.275	15.969–164.643	< 0.001	1.502	0.266–8.473	0.645
Omental cake	Yes vs No	6.897	4.588–10.368	< 0.001	0.803	0.422–1.529	0.505
Hepatic hilum involvement	Yes vs No	19.802	10.035–39.075	< 0.001	1.950	0.711–5.348	0.195
Mesenteric involvement	Yes vs No	6.589	4.402–9.862	< 0.001	2.006	1.215–3.312	0.007
Small omentum involvement	Yes vs No	19.484	9.873–38.451	< 0.001	0.805	0.277–2.342	0.690
Abdominal mass	Yes vs No	5.920	3.496–10.024	< 0.001	0.567	0.230–1.399	0.219

Note: ^*a*^*CI*, confidence interval; ^b^*US-CT PCI*, peritoneal cancer index evaluation by combining ultrasonography and computed tomography

## Discussion

For most peritoneal metastasis types, S-PCI emerged as an independent factor associated with survival outcomes [15] and high PCIs (≥ 20) were occasionally considered the criterion for the resectability of PMP [21]. This study was the first to document the correlation between US-CT and surgical findings. We showed that US-CT PCI could help predict the possibility of complete CRS. Shigeki Kusamura et al [22] obtained results that are consistent with this conclusion. Therefore, preoperative imaging was suggested imperatively in order to select patients that should undergo CRS appropriately. CT was good at describing both location and morphology of the mucocele [23, 24]. However, CT PCI was underestimated in peritoneal spread evaluation due to the partial volume effect [15]. MRI was more sensitive at identifying whether the mucocele is mucin- or fluid-filled due to superior contrast resolution. Engbersen, MP et al [25] demonstrated that MRI-PCI allowed to accurately predict S-PCI due to the better detection of small peritoneal lesions (< 5 mm) than CT. Also, MRI may provide more information in bowel involvement assessment [8, 9, 11]. Meanwhile, US had potential advantages in distinguishing the boundary of mucinous particles, especially those with a diameter smaller than 0.5 cm, which was obviously better than CT [14]. In addition, the report also showed the wall shear stress and blood flow volume recorded in the superior mesenteric artery had efficacy in predicting PMP progression [26, 27].

However, it was difficult for one single diagnostic method to reflect disease burden with high accuracy. Hence, approaches combining parameters (CT and US) possessed obvious advantages. Besides, a study on pelvic mass diagnosis reported that US-CT yielded a correct preoperative diagnosis, helping patients with treatment decisions [28]. In the current study, US-CT PCI accurately reflected tumor progression. We performed US-CT based on the relatively high accuracy of US to quantify PMP tumor burden in the nine abdominal regions and CT in the four small bowel regions. Although small bowel involvement may sometimes be difficult to detect by CT, CT still had higher diagnostic accuracy than US (*p* < 0.001), which may be explained by the lack of landmarks to correctly identify the four sections by US. Besides, we noted a gap in performance between CT and US in R4 and R5 but not in R7 and R8. One explanation could be that the appendix lesions were usually located in R7 and R8; thus, the two imaging methods had similar detection rates.

However, tumor with a high PCI may still be amenable to complete excision and cure. Additionally, a tumor on the serosa of the small bowel, or at the junction of the small bowel and its mesentery, was difficult to surgically excise and precluded complete tumor removal [29]. The diagnostic challenge with PMP was identifying resectable versus unresectable tumors. The present study identified mesenteric involvement as a poor prognostic factor. Results from Mittal, R et al [12] supported that the involvement of crucial anatomic sites such as small bowel surface or its mesentery was related to cytoreduction possibility. Small bowel involvement inevitably led to intestinal failure and poor quality of life [29], which may explain its dominant prognostic determinants of cytoreduction feasibility. This study also suggested that the male sex was independently associated with debulking surgery, which was supported by Bai, M et al [30], who noted indolent behavior and nonspecific symptoms in males. Male patients with PMP were often diagnosed at advanced stages and with gradually increasing abdominal circumference, abdominal distension, bowel obstruction, ascites, or new onset hernia. In contrast, female patients could be diagnosed incidentally through pelvic imaging studies for existing symptoms or ovarian masses [31]. Thus, although PMP was more common in females (4:1), females often tended to be diagnosed earlier and therefore had a higher resectability [12].

This study presented several limitations. First, although the cohort was quite large for this rare disease, the study was still a single-center study. Second, we lacked the resources to collect MRI data and evaluate MRI PCI. We will collect and study more relevant data in the future.

To conclude, preoperative imaging with combined US and CT improved the diagnostic accuracy of PCI. And imaging features detected by US-CT could help select patients for CRS.

## Supplementary Information

ESM 1 (DOCX 988 kb)

## Abbreviations

CCR: Completeness of cytoreduction
CRS: Cytoreductive surgery
CT: Computed tomography
HIPEC: Hyperthermic intraperitoneal chemotherapy
HR: Hazard ratio
MRI: Magnetic resonance imaging
PCI: Peritoneal cancer index
PMP: Pseudomyxoma peritonei
US: Ultrasonography

## Declarations

### Guarantor

The scientific guarantor of this publication is Lei Liang.

### Statistics and biometry

No complex statistical methods were necessary for this paper.

### Methodology

• retrospective

• diagnostic or prognostic study

• performed at one institution
