# Supplementary figures and images for: Combined ultrasonography and CT for prognosis and predicting clinical outcomes of patients with pseudomyxoma peritonei
Source: Eur Radiol. 2022 Nov 23;33(4):2800–8. doi: 10.1007/s00330-022-09242-z (PMC10017557; doi:10.1007/s00330-022-09242-z)

Supplementary Figure 1. The learning curve of PCI evaluation.


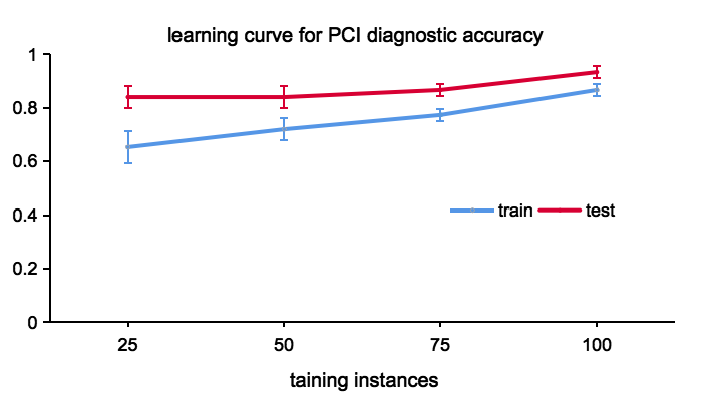

Supplement: Supplementary file 1 — (DOCX 988 kb) [file 330_2022_9242_MOESM1_ESM.docx]
